# Cuticles of European and American lobsters harbor diverse bacterial species and differ in disease susceptibility

**DOI:** 10.1002/mbo3.174

**Published:** 2014-05-12

**Authors:** Miranda M A Whitten, Charlotte E Davies, Anita Kim, Michael Tlusty, Emma C Wootton, Andrei Chistoserdov, Andrew F Rowley

**Affiliations:** 1 Department of Biosciences, College of Science, Swansea UniversitySingleton Park, Swansea, SA2 8PP, U.K; 2 John H. Prescott Marine Laboratory, New England AquariumCentral Wharf, Boston, Massachusetts, 02110; 3 Department of Biology, The University of Louisiana at LafayettePO Box 42451, Lafayette, Louisiana, 70504

**Keywords:** *Aquimarina*, biofilm, emerging infectious diseases, invertebrate microbiology, lobster pathobiology, shell disease syndrome

## Abstract

Diseases of lobster shells have a significant impact on fishing industries but the risk of disease transmission between different lobster species has yet to be properly investigated. This study compared bacterial biofilm communities from American (*Homarus americanus*) and European lobsters (*H. gammarus*), to assess both healthy cuticle and diseased cuticle during lesion formation. Culture-independent molecular techniques revealed diversity in the bacterial communities of cuticle biofilms both within and between the two lobster species, and identified three bacterial genera associated with shell lesions plus two putative beneficial bacterial species (detected exclusively in healthy cuticle or healing damaged cuticle). In an experimental aquarium shared between American and European lobsters, heterospecific transmission of potentially pathogenic bacteria appeared to be very limited; however, the claws of European lobsters were more likely to develop lesions when reared in the presence of American lobsters. Aquarium biofilms were also examined but revealed no candidate pathogens for environmental transmission. *Aquimarina* sp. ‘homaria’ (a potential pathogen associated with a severe epizootic form of shell disease) was detected at a much higher prevalence among American than European lobsters, but its presence correlated more with exacerbation of existing lesions rather than with lesion initiation.

## Introduction

The American lobster, *Homarus americanus* (Milne Edwards, 1837), has been considered an invasive species in European waters since the first genetically confirmed cases were discovered off Norway in 1999 (Jørstad et al. 2007). The escape or release of live imported American lobsters is the most likely method of introduction of this species. There have been 26 reports of American lobsters in British waters since 2010 (Moore et al. 2011).

Invasive *H. americanus* can be found throughout most of the native range of the European lobster, *Homarus gammarus* (Linnaeus, 1758) (van der Meeren et al. 2010; Stebbing et al. 2012). The invasion could present a risk via competition, dilution of genetic stock by hybridization, or disease transmission. Of concern is the finding that a small number of American lobsters trapped in Norwegian waters have exhibited symptoms of epizootic shell disease (ESD; Karlsbakk et al. 2011), with lesions containing bacteria associated with ESD (A.-L. Agnalt, pers. comm.). This disease manifests as severe coalescing lesions and is responsible for significant economic losses to American lobster fisheries (Castro et al. 2006). While no published reports exist of ESD among European lobsters to date, it remains unknown whether *H. gammarus* is capable of contracting this particular form of shell disease, and whether it would manifest in the same manner as seen in *H. americanus*.

Other forms of shell disease, such as enzootic (or ‘classic’) shell disease, are commonly observed among both American and European lobsters. Most forms of shell disease are believed to have a primarily bacterial etiology (e.g., Chistoserdov et al. 2012), although fungi and other microbes are also implicated (reviewed in Vogan et al. 2008). The causes of ESD and other forms of crustacean shell disease are not well understood but there is a general consensus that such diseases arise from a combination of multiple biotic and abiotic stressors (such as poor diet, temperature or pollution) that increase a lobster's susceptibility to environmental bacteria (e.g., Tlusty et al. 2007, 2008; Castro et al. 2012). It has also been hypothesized that shell disease has a polymicrobial basis during a ‘dysbiotic shift’ of the normal flora (Bell et al. 2012; Meres et al. 2012). The identity of the bacteria responsible for ESD is still a matter of considerable debate, but recent studies highlight the association with Rhodobacteriaceae (*Thalassobius* sp.), flavobacteria (*Aquimarina* sp. ‘homaria’*, Maribacter* sp.) and *γ*-proteobacteria (*Pseudoalteromonas* sp.), with many more exacerbating the condition (Chistoserdov et al. 2009, 2012; Quinn et al. 2012). The multifactorial and possibly overlapping etiologies of ESD and other shell diseases not only make them difficult to study but also may confound a clear diagnosis, particularly as one form of shell disease might act as a precursor for another (Quinn et al. 2012; Tlusty and Metzler 2012; Smolowitz et al. 2013). Laboratory experiments have failed to demonstrate transmission of ESD between American lobsters (Chistoserdov et al. 2005) while heterospecific transmission of shell disease and ESD has not yet been investigated. However, a form of shell disease was found to be successfully initiated in captive American lobsters by abrading the cuticle (Quinn et al. 2012). In this case, the source of the causative bacteria was probably waterborne, though biofilms have not been ruled out as a source for pathogenic bacteria.

Injury is a probable precursor to many forms of shell disease, since it exposes underlying layers and soft tissue. Therefore, just as a bacterial component is integral to shell disease pathology, injury is a very important risk factor. In confined spaces (including traps), the risk of aggressive behavior, injury, and disease transmission is at its highest. In particular, common injury sites are the sides of the dorsal carapace and the dorsal tail fan (usually abrasion) and the propus and dactyl regions of the claw (deep punctures and cracks). The severity of such injuries and damage is dependent on the density of the lobster population (Wootton et al. 2012).

In the current aquarium-based comparative study, the cuticle of subadult American and European lobsters was experimentally breached to simulate the types of injury commonly inflicted during self-abrasive behaviors and during fighting. This provided a mechanism with which to follow the time course and incidence of subsequent lesion formation, to observe changes in the bacterial community of the cuticle both before and during shell disease development, and to compare the cuticular microflora of the two lobster species. Additionally, culture-independent molecular methods were used to identify bacterial species either associated with initiating lesions as potential pathogens, or potentially beneficial species associated with healthy or healing cuticles. Differences in the microflora of damaged cuticles might provide insight into susceptibility to shell disease and ESD. By comparing European lobsters held in a communal aquarium shared with American lobsters, with a control group of European lobsters held in a separate aquarium from which American lobsters were absent, the study also aimed to identify any gross changes in lesion progression and/or associated bacteria that might suggest heterospecific shell disease transmission. Biofilm samples from the tank walls of both aquaria were also studied to determine the possible role of the environment as a source of bacterial infection. Finally, the presence of the flavobacterium, *Aquimarina* sp. ‘homaria’, was investigated using molecular methods to shed further light on its role as a possible shell disease pathogen.

## Experimental Procedures

### Lobster rearing and transport

Juvenile European lobsters (*H. gammarus*) were reared from eggs in a disease-free environment at the Centre for Sustainable Aquatic Research, Swansea University, UK. All the lobsters originated from the same hatching batch. Upon reaching a minimum carapace length (CL) of 34 mm, the animals were divided into two groups (of 11 and 12 individuals) and the first group was transported overnight in chilled containers by Wilder Logistics Ltd., UK, to the Lobster Research and Rearing Facility at the New England Aquarium, Boston, USA. Upon arrival, the lobsters were housed, unbanded, in individual plastic containers (13.5 × 21 × 13 cm) (to prevent fighting) within a communal aquarium tank that was shared with a group of 10 juvenile American lobsters (*H. americanus*), which were also housed individually in plastic containers (10 × 22 × 9 cm). The American lobsters were reared from eggs, at the Lobster Research and Rearing Facility. The communal tank operated within an 1132 L semiclosed recirculation system with water from Boston Harbor. The two species groups remained in the same tank and were exposed to the same environmental conditions without making direct physical contact, from the time of receipt until the end of the experiment. The second group of European lobsters remained in the United Kingdom and were chilled in the dark to control for transportation stress, and then maintained in Swansea University's main aquarium (an enclosed re-circulating system of natural seawater from Swansea Bay) for 2 months prior to the start of the experiment and for the duration of the experiment. The size of the American lobsters ranged from 35 to 45 mm CL, and the European lobsters ranged from 34 to 75 mm CL (for further details, see Table S1). The European lobsters shipped to the United States were selected to be of a size as close as possible to the experimental American lobsters, thus allowing for better comparison of the two species held in the Boston location. Irrespective of their location, the lobsters' claws were left un-banded, and all the animals were fed thrice-weekly on a gel-based diet (Tetra marine mix gel, Spectrum Brands, Melle, Germany, and Mazuri® gel diet, PMI Nutrition International LLC, St. Louis, MO); the lobsters in Swansea additionally received fresh *Mytilus edulis* mussels.

### Abrasion/impact damage experiments

Lobster shell damage was mechanically induced by abrading the waxy, non chitinous epicuticle of the carapace and by introducing deeper impact damage via cracks or punctures on the claw. An area ca. 10 mm^2^ on the dorsolateral carapace was abraded with ultraviolet-irradiated 400-grit wet/dry sandpaper (3M). Areas were abraded for 30 sec (European lobsters) and 20 sec (American lobsters) to expose the chitinous inner exocuticle layer. These time differences reflected variations in the cuticle texture, with less abrasion required to reach the same depth in American lobsters. The opposite side of the carapace was left unabraded as a control region. Experimental lobsters additionally received either a simulated crack injury or a small puncture to the dorsal propus of the claw by lightly tapping a sterile flathead or Phillips screwdriver into the cuticle (with ∼5 mm head length/diameter). The control area was on the opposite claw (unless missing, in which case another site on the dorsal propus was chosen).

Anatomically, the damaged sites were located identically on all lobsters, unless the area exhibited preexisting damage, spotting or abnormal pigmentation. An unaffected area nearby was chosen in these cases. The lobsters in this study were abraded and damaged at varying time points during their molt cycles (time from one molt to the next); in most cases ranging between 17 and 100 days post molt the start of the experiment (time zero), and a few individuals with unknown intervals exceeding 100 days). For further details, see the Data analyses section below, and Table S1.

### Whole-body cuticle health survey

For the 2 months preceding the study, during, and for several weeks after its conclusion, the entire cuticle surface of lobsters was examined and photographed fortnightly to provide an overall indication of the cuticular health status. Full details are in the Data S1.

### Imaging

Low-magnification images were captured using Nikon Coolpix (Nikon UK Ltd, Kingston Upon Thames, Surrey, UK) and Canon Powershot G12 digital cameras (Canon USA, Inc., Melville, NY). High-magnification images of the cuticle were captured using a Daray LCD microscope (Daray Ltd, Moira, UK). Image modification was limited to cropping and minor adjustments of exposure and contrast to compensate for heavy background pigmentation (Preview v.5.0.3, Apple Inc., Cupertino, CA).

### Surveillance of environmental and cuticular bacterial biofilm communities

To determine how microbial communities on the shell might change over time in both healthy and damaged shell areas, all test and control areas on the lobsters were photographed and swabbed with sterile cotton swabs before the experiment to assess the biofilm bacterial microflora. A longitudinal study incorporating a systematic fortnightly sampling routine was then employed for the next 10 weeks, in which damaged areas were recorded and photographed, and samples of damaged and control cuticular material were preserved for later molecular analysis of microbial communities. A sterile swab was used to transfer the material onto FTA® cards (Whatman International Ltd, Maidstone, UK), and swabs were also preserved in 95% ethanol at −20°C. It was unknown whether repeated sampling would alter the natural formation of microbial communities over time or alter the progress of shell disease. Therefore, half of the lobsters, for each species and in each location, were sampled at each fortnightly time point (American lobsters *n* = 5; European lobsters in the United States *n* = 6; European lobsters in the United Kingdom *n* = 6), while the remaining lobsters were sampled after initial damage and again at the end of the experiment (American lobsters *n* = 5; European lobsters in the United States *n* = 5; European lobsters in the United Kingdom *n* = 6).

At the start and end of the experiment, swabs were also taken of tank biofilms, individual lobster containers, and from filter-concentrated aquarium water samples.

### DNA extraction

The bacterial community of biofilm samples from lobster healthy, damaged and lesioned cuticle, and from the aquarium tank environment, was examined using culture-independent molecular methods. Several DNA extraction methods were evaluated for the preserved biofilm samples (see Data S1 for details). The optimal method was from ethanol-preserved swabs, using a modification of the Qiagen DNeasy® Blood and Tissue Kit (Qiagen Ltd, Manchester, UK) incorporating an additional initial lysozyme-based disruption step optimized for Gram-positive bacteria, followed by proteinase K digestion (see Data S1). The manufacturer's protocol was then followed and DNA was eluted with water and used as the template for subsequent touchdown polymerase chain reaction (PCR) to amplify 16S rRNA (see below).

### PCR-TTGE

Bacterial communities were profiled using the PCR-temporal temperature gradient gel electrophoresis (TTGE) method. Seventy-one cuticle swab samples were compared by TTGE to identify novel bands that either disappeared from healthy cuticle during lesion progression, appeared for the first time in spots or lesions, or were associated with samples testing positive for *A*. sp. ‘homaria’. Eleven environmental samples were also similarly analyzed. Touchdown PCR was used to amplify bacterial 16S rRNA. Cycling conditions were: 2 min at 93°C followed by 37 cycles of 1 min at 93°C, 30 sec at 60°C (−0.5°C/cycle for the first 10 cycles and 55°C thereafter), and 1 min at 72°C, followed by 6 min at 72°C. Following preliminary evaluations of several candidate primer pairs (criteria: diversity of coverage, replicable band separation, and migration by TTGE), the primers PRBA338f and PRUN518r were selected to target universal zones flanking the hypervariable V3 region of the bacterial 16S rRNA gene (Øvreås et al. 1997), and these primers were designed to reduce the risk of chimera formation during amplification. PRBA338f: 5′-ACTCCTACGGGAGGCAGCAG-3′ (Lane 1991). PRUN518r: 5′-ATTACCGCGGCTGCTGG-3′ (Muyzer et al. 1993). A GC clamp was attached to the 5′ end of PRBA338f to prevent total strand dissociation during electrophoresis: 5′-CGCCCGCCGCGCGCGGCGGGCGGGGCGGGGGCACGGGGGG-3′ (Muyzer et al. 1993). Amplifications were performed with Mango mix reagents (Bioline Reagents Ltd, London, UK). Single-species bacterial DNA templates were included as positive controls and the reproducibility of the PCR reaction and resulting TTGE banding patterns were confirmed by running several replicates, with highly reproducible results.

### TTGE

Amplicons from the above PCR reactions were separated by TTGE using the BioRad DCode™ Universal Mutation Detection System (BioRad Laboratories Inc., Hemel Hempstead, UK) with 7% polyacrylamide gels supplemented with 7 mol/L urea and 2% v/v glycerol. The optimized running temperature ramped from 49.3°C to 68.5°C at a rate of 2.7°C/h. The running buffer was 1.43× Tris-Acetic acid-EDTA buffer. Generic size markers (100 bp DNA ladder, New England Biolabs Inc., Hitchin, UK), were included at either side of each gel to detect skew and facilitate subsequent band alignment. The gels were post stained with GelRed™ (Biotium, Hayward, CA) and analyzed visually and by GelAnalyzer software (v. 2010, http://www.gelanalyzer.com, Istvan Lazar, Hungary, for standardizing band migration coefficients between gels) to assess banding patterns as a gauge of bacterial diversity, relative abundance, and distribution. It was of particular interest to identify any bands that either appeared in, or disappeared from, the majority of damaged cuticles during lesion formation or during healing, indicating (respectively) possible pathogens or beneficial bacteria. Healing was defined as the sealing of cuticle damage and exposed areas of underlying tissues, often accompanied by melanization but without signs of infection and necrosis. Over 25 TTGE gels were run, from which bands of particular interest, representing individual bacterial species, were excised on an ultraviolet transilluminator for subsequent cloning into the pGEMTeasy cloning vector (Promega). Details of the cloning protocol are provided in the Data S1.

### Sequencing and phylogenetic analysis

Minipreps of the cloned bands-of-interest were sequenced using M13-21F and R universal primers (LGC Genomics, Berlin, Germany) and the resulting consensus sequences were screened using the DECIPHER online analysis tool to confirm the absence of potential chimeras (http://decipher.cee.wisc.edu/FindChimeras.html; Wright et al. 2012). These sequence data have been submitted to the National Center for Biotechnology Information (NCBI) GenBank database under accession numbers KF631432 to KF631443. Sequences were then analyzed using Clustal Omega (for multiple sequence alignment, http://www.ebi.ac.uk/Tools/msa/clustalo/) and the sequences were aligned to 16S rRNA sequences obtained from NCBI GenBank database using programs within the Ribosomal Database Project II (RDP II, http://rdp.cme.msu.edu; Cole et al. 2005), and NCBI BLAST search. ClustalW2 was used for basic phylogram construction to create a neighbor-joining tree (see Fig. 5), which was edited in CTree (v.1.0; Archer and Robertson 2007).

### PCR-based detection of *Aquimarina* sp. ‘homaria’

Biofilm DNA extracts were screened for *A*. sp. ‘homaria’ using a species-specific PCR assay. Samples were analyzed from (1) healthy carapace, (2) damaged but healing carapace, (3) at regular time points during induced lesion formation, and (4) during natural lesion formation on other parts of the lobster shell. The assay employed the specific primers Ah190F (5′- TAGTATCMAAGACAGCMTTGTTTTATG-3′) and Ah470R (5′-CCTTATTCGTAGAGTACCGTCAGAGTAT-3′) and the method of Chistoserdov et al. (2012) with the following modifications incorporating touchdown conditions: 2 min at 95°C followed by 35 cycles of 15 sec at 95°C, 30 sec at 62°C (−0.5°C/cycle for the first 24 cycles and 50°C thereafter), and 30 sec at 72°C, followed by 3 min at 72°C. Randomly chosen amplicons were sequenced to confirm the specificity of the PCR reaction. Additionally, the PCR reaction was found to be unusually sensitive to template concentration, and therefore any negative samples were verified by performing extra PCR amplifications using a template concentration gradient (10 pg to 100 ng per 50 *μ*L reaction). The specificity of the reaction was checked by obtaining consistently negative reactions with 23 templates from type strain isolates of other bacterial species.

### Data analyses

All statistical analyses were performed using Prism v.4 and v.6 (Graphpad Software, Inc., San Diego, CA). Differences between the prevalence of *A*. sp. ‘homaria’ among American and the two European lobster populations were analyzed by chi-square analysis (three-way analysis for individual populations and with Fisher's exact test for two-way analysis grouping the European lobsters). Differences in the time course of lesion formation, expressed as a cumulative percentage of affected animals, was assessed by the Kaplan–Meier survival analysis followed by the log rank test. This analysis automatically adjusts for censored subjects (i.e., the cumulative percentage accounts for animals dropping out of the study before its conclusion due to molting or a single case of mortality, and for subjects being recruited to the study late due to the cuticle being too soft to abrade after a recent molt). Molt intervals (i.e., the time elapsed since the last molt) were not able to be controlled due to experimental time constraints. Spearman correlation analyses were used to determine relationships between a lobster's size or its stage in the molt cycle and its risk of lesion development. These relationships are explored fully in the Data S1 and Figures S1–S3.

## Results

### Lesion formation on experimentally damaged cuticle

For both lobster species and in both aquarium locations, abrasion of the carapace and impact damage to the claw resulted in the formation of a melanin response within 2–3 days. Over the next 8–10 weeks, several lobsters developed damage-associated lesions, which were typically more severe on the claws than on the carapace.

**Figure 1:**
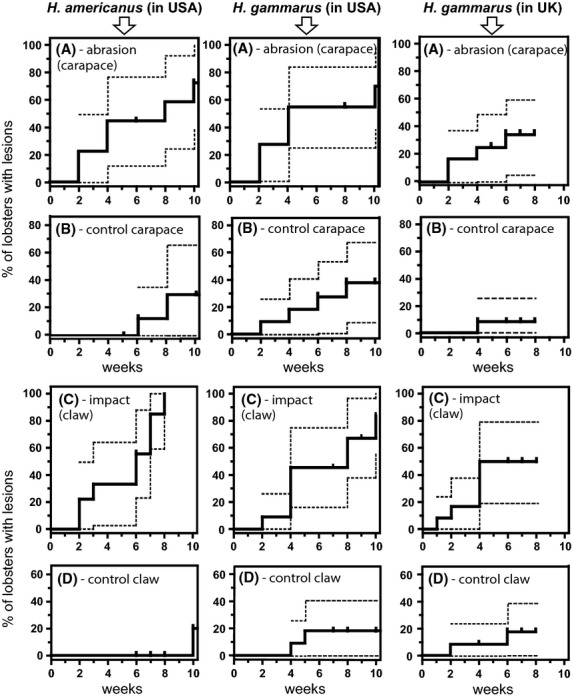
Lesion formation on experimentally abraded lobster carapace and damaged claw. The time course and prevalence of new lesion formation on (A) experimentally abraded carapace or (C) experimentally damaged claw, versus the spontaneous appearance of new lesions in corresponding regions of (B) the control carapace or (D) control claw, in American and European lobster cohorts held in Boston (USA) and Swansea (UK). Data for the two types of damage (crack, puncture) have been pooled. Kaplan–Meier survival curves showing 95% CI (dotted line). Lobsters that were too soft from a recent molt were added to the experiment after a delay to allow cuticle hardening (indicated by vertical ticks as “censored participants”).

By 8 weeks post damage, there was a consistent increase in the number of lesions that formed on abraded carapace compared with control regions for each lobster species and aquarium. The cumulative percentage of lobsters exhibiting lesions on the abraded carapace versus control carapace region, was, respectively: 58% and 22% of American lobsters (held in Boston, USA), 55% and 36% of European lobsters (held in Boston, USA), and 34% and 8% of European lobsters (held in Swansea, UK) (Fig. 1A). It should be noted that animals entered and exited the study at different time points (some were recruited late to allow cuticle hardening after a recent molt, some dropped out before 8 weeks due to molting and one lobster died at 7 weeks post abrasion). It is, therefore, more accurate to assess lesion formation by survival analysis, as shown in Figure 1A and B (see Experimental Procedures: Data analyses, for details). Although there was an increased occurrence of carapace lesions following induced abrasion compared with control regions of the carapace, damage was not correlated with an increased risk of lesion formation on the carapace (*P* > 0.05, log rank test). Additionally, lesion prevalence for abraded carapace samples was not significantly influenced by either the lobster species or its aquarium location (Fig. 1A; Kaplan–Meier survival analysis followed by log rank test, *P* > 0.05). Importantly, this means that there were no significant differences between the prevalence of carapace lesion formation between the two European lobster populations despite being exposed to different microbial environments (see section on Bacterial community analysis by TTGE below).

On the claw regions, however, damage was strongly correlated with an increased risk of lesion formation, but only for lobsters held in the United States. The cumulative percentage of lobsters exhibiting lesions on damaged claw and claw controls were, respectively, 100% and 0% of American lobsters, 67% and 18% of European lobsters held in Boston, USA, and 50% and 17% of European lobsters held in the United Kingdom (Fig. 1C and D). American and European lobsters held in the United States were significantly more likely to develop a claw lesion during the experiment after impact damage, compared with the control claw regions (*P* < 0.0001 for American lobsters and *P* < 0.05 for European lobsters held in the United States; log rank with chi-squared test, df = 1), but this was not significant in European lobsters held in the United Kingdom.

The incidence of spontaneous (uninduced) lesions occuring on either the carapace (Fig. 1B) or claw regions (Fig. 1D) during the course of the study was not significantly different between the lobster species or aquarium location (*P* > 0.05, chi-squared tests, df = 2).

However, no correlation existed between the molt interval and the risk of lesion formation on either the experimentally damaged cuticle or on the undamaged control regions, and nor was a correlation found between lobster size and the risk of lesion formation during the experimental period (Spearman correlation analyses; see Data S1 for details).

**Figure 2:**
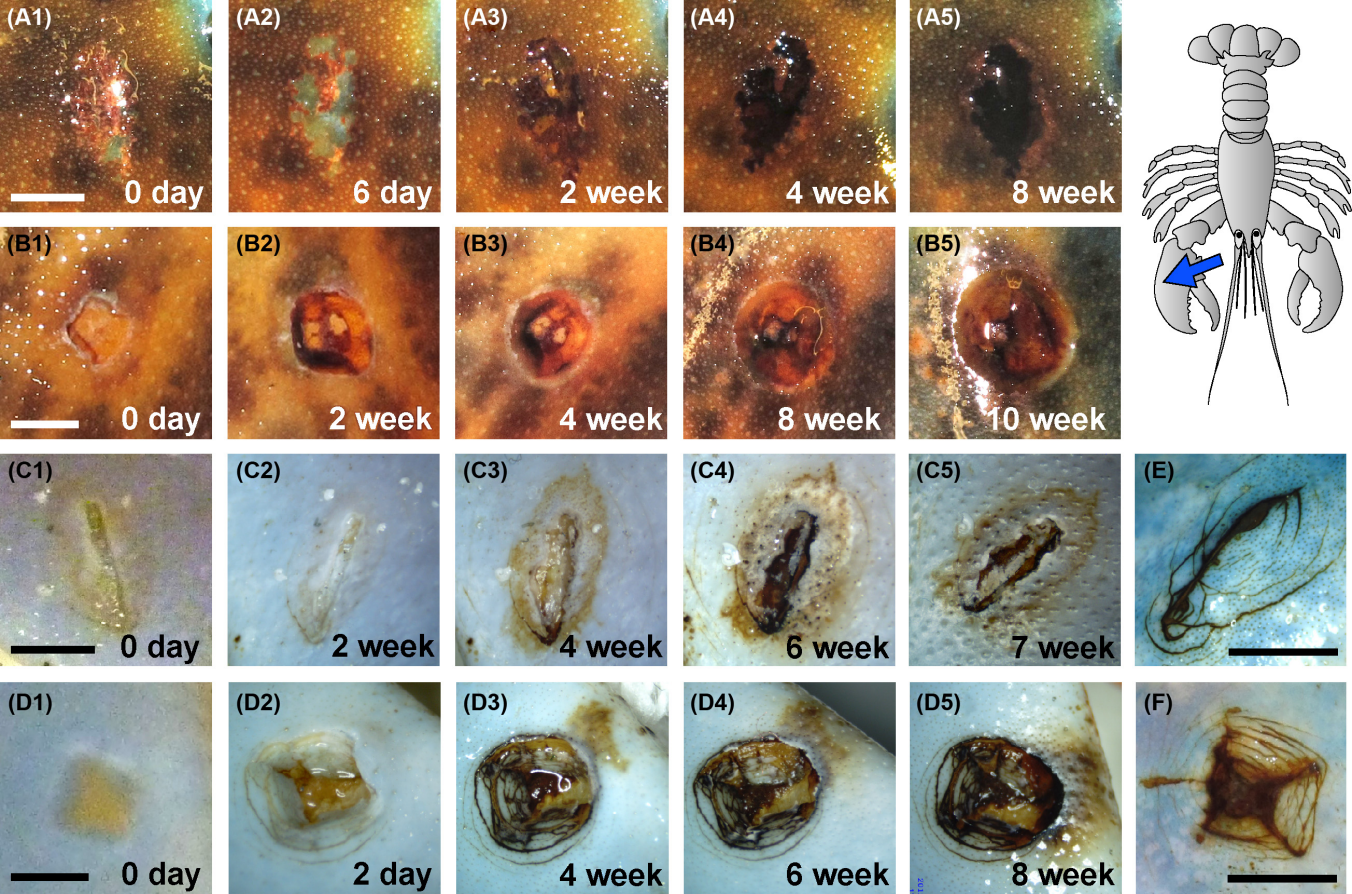
Examples of progressive necrosis and lesion formation on experimentally damaged claw cuticle from American and European lobsters. Top row (A1–A5) = cracked American lobster claw propus, over 8 weeks. Second panel (B1–B5) = punctured American lobster claw propus, over 10 weeks. Third row (C1–C5) = cracked European lobster claw propus, over 7 weeks. Fourth row (D1–D5) = punctured European lobster claw propus, over 8 weeks. Healed European lobster cuticles are shown for comparison (E = cracked; F = punctured; no healed examples exist for the American lobster cohort). No obvious differences were noted in common lesion morphologies between the two European cohorts (examples shown here are from the UK aquarium). Brightness and contrast settings have been adjusted to compensate for dark background pigmentation. Bars = 5 mm (images at later timepoints are all to the same scale).

**Figure 3:**
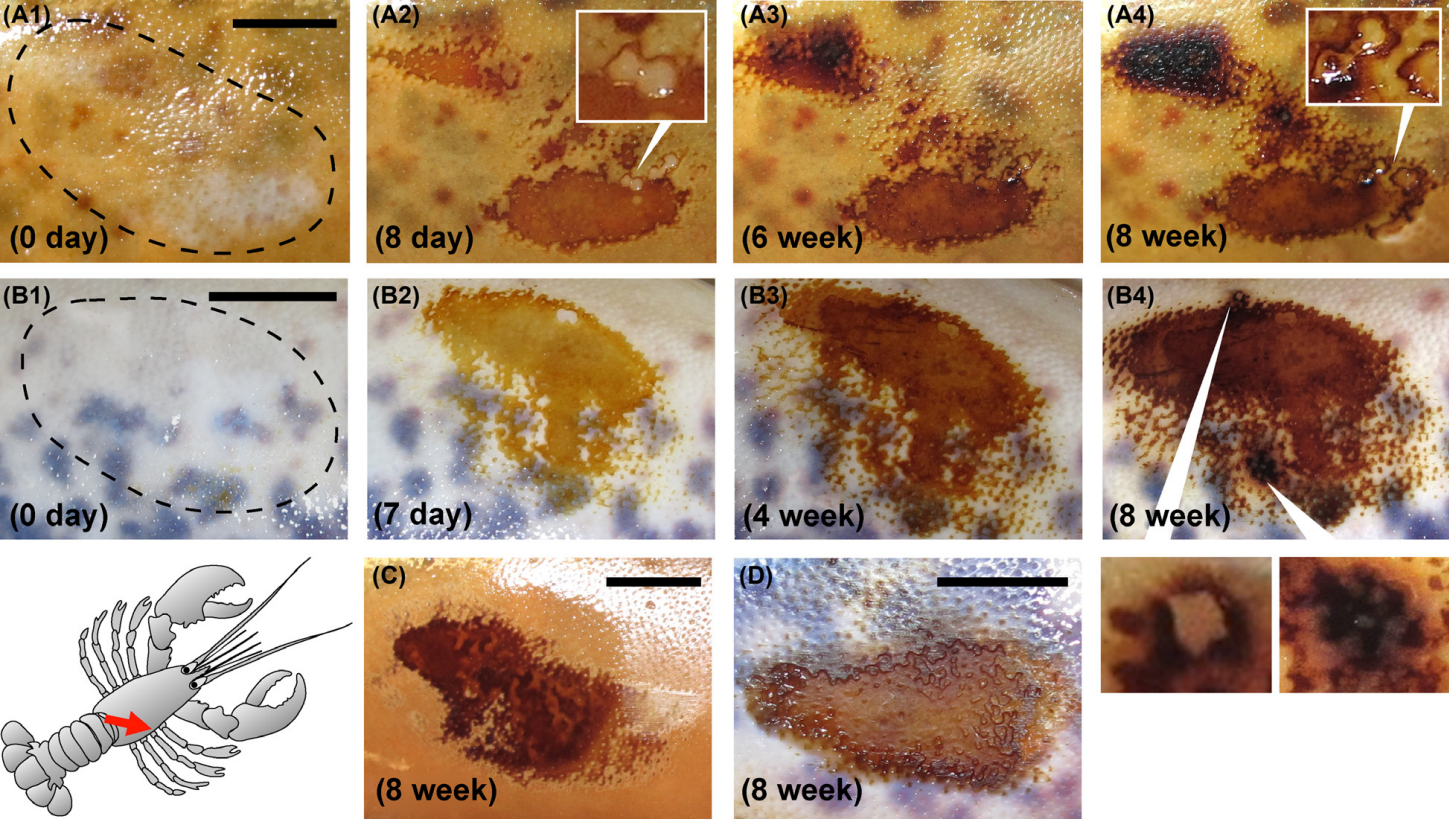
Examples of progressive necrosis and lesion formation on experimentally abraded carapace from American and European lobsters. Top row (A1–A4) = American lobster carapace, over 8 weeks. Second row (B1–B4) = abraded European lobster carapace, over 8 weeks. Note the small pitted lesions and raised hypermelanotic areas (inset boxes). Experimentally induced carapace lesions were typically less severe than damaged claw lesions. No obvious differences were noted in common lesion morphologies between the two European cohorts. Healed cuticles are shown for comparison (C = American; D = European). Bars = 5 mm (images at later timepoints are all to the same scale). The time post abrasion is indicated in parentheses. The freshly abraded areas are indicated with a dashed black line at 0 days.

In most cases, and in both lobster species, the severity of lesions on the damaged claw exceeded that of carapace abrasions (see examples in Figs. 2, 3). While a small percentage of the abraded carapace area became necrotic (typically less than 25% of the total area), it was common for the entire area of damaged claw to become necrotic (i.e., exhibiting dead, discolored cuticular tissue).

Molting cannot be controlled for, but it is obviously one valid mechanism by which a lesion can be resolved. There were cases of individual lobsters molting in each of the cohorts (between one and three animals in each group; see Table S1 for details), and molting always occurred in the late stages of the experiment. One lobster died (a European lobster held in the United Kingdom), and this occurred within a week of the conclusion of the experiment.

### Lesion morphologies

The experimentally induced lesions predominantly displayed two distinct morphologies overall. One form expanded uniformly from the peripheral circumference, creating a wide, shallow lesion with smooth edges (e.g., Figs. 2B1–B5, 3A1–A4). This was associated with puncture wounds on the claws and abrasion damage on the carapace. However, this morphology was notably absent from the experimentally damaged claw areas of European lobsters held in the United Kingdom (see Fig. S4). A second form of lesion, more commonly seen with impact damage to the claws (and especially in European lobsters held in the United Kingdom, see Fig. S4), had characteristically irregular, rough edges, with erosion not only continuing outward on the surface but also underneath, undermining the superficial layers of the cuticle (e.g., Fig. 2C1–C5). This latter form resulted in deep lesions with pale, discolored crusts, often with asymmetric melanotic discoloration leaching outward from the lesion periphery which was considered necrotic (e.g., Fig. 2C and D). Some areas of abraded carapace and damaged claw exhibited more than one form of lesion, and occasionally these were accompanied by regions of excessively melanized, raised material (Fig. 3B4). Healed cuticular damage, in contrast, melanized rapidly with no evidence of discoloration or expanding peripheries (e.g., Figs. 2E and F, 3C and D) and was similar in both lobster species in both locations. One animal (European lobster EU026, held in the United States) exhibited exceptionally severe abraded carapace lesions. This animal concomitantly developed additional spontaneous lesions over much of its dorsal carapace (for further details, see Fig. S5).

### Bacterial community analysis by TTGE

**Figure 4:**
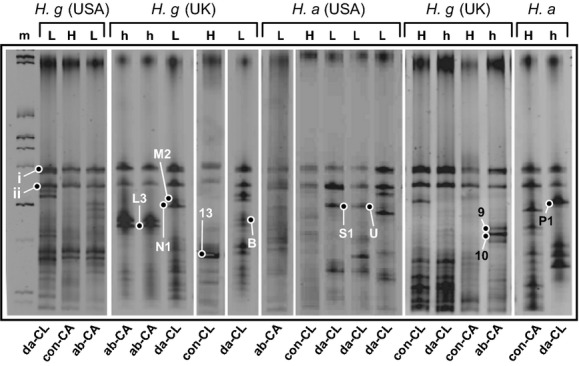
Examples of TTGE gel banding patterns for bacterial 16S rDNA amplified from lobster cuticle samples. Claw (CL) and carapace (CA) samples from American lobsters (*Homarus americanus*) and European lobsters (*Homarus gammarus*) are included. Various interventions are indicated as follows: da = impact damage (crack or puncture); ab = abrasion; con = control (undamaged). The health of the cuticle is indicated by: L = lesion; H = healthy undamaged; h = healed damage. Several bands-of-interest are indicated, which were chosen for sequencing (see also Table 1); these bands were consistently associated with particular health statuses across several lobster samples. Two bands (i and ii) were always present irrespective of the sample. m = marker lane (markers on either side of each gel were used for band alignment; sizes are not indicated since nucleotide separation is sequence-dependent). Composite image from several independent gels.

Bacterial species, detectable as discrete bands by TTGE, were present in every environmental and cuticular swab tested (example gels are shown in Fig. 4). The overall mean number of bacterial species from healthy cuticle samples was 8.4 per swab (±SE 0.3) and did not vary significantly between lobster species, aquarium location, or anatomical region (all comparisons *P* > 0.05, one-way analysis of variance [ANOVA] with Tukey's post test). Compared with healthy samples, the bacterial diversity in lesioned samples among all the lobster cohorts increased significantly, rising to a combined mean of 11.1 species (±SE 0.7). Diversity was highest at the time of initial lesion formation (*P* = 0.031, paired *t*-test, df = 6).

Cuticular bacterial communities exhibited diversity between individuals, between different anatomical regions, and between different time-points of the same anatomical region. Two bands were present in virtually every sample, irrespective of origin, and probably represent highly ubiquitous bacterial species not directly relevant to cuticle health; these species were not investigated further (Fig. 4, bands i and ii). Many other bands were present, irrespective of the health of the cuticle, but there were few commonalities between samples. No banding patterns were identified that could be considered characteristic of each group of lobsters (i.e., the number and distribution of bacterial species; detected as discrete bands, did not correlate with lobster species). Lesion morphologies were not obviously associated with particular bacterial flora. The proximity to American lobsters had no significant impact on overall bacterial diversity among the European lobsters (one-way ANOVA with Bonferroni's multiple comparisons test; *P* > 0.05 for each cuticular sample; initial and final time points assessed). Thus, the cuticle-associated bacteria from European lobsters were equally diverse in each of the two separate aquarium systems (United States and United Kingdom).

**Figure 5:**
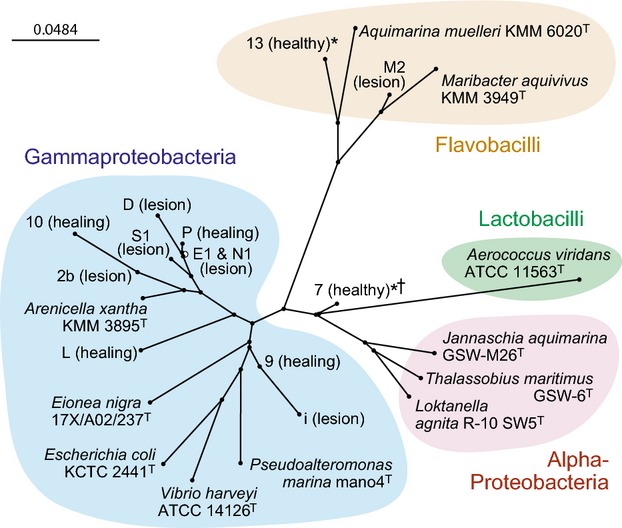
Basic phylogenetic tree of bacterial partial 16S rRNA gene sequences constructed using the ClustalW2 neighbor-joining method. The tree shows sequences from selected TTGE bands from lobster cuticle samples (numbers/letters, with health status of the cuticle indicated in parentheses). Stars (*) indicate species that disappear from healthy cuticle upon lesion formation; dagger (†) indicates a short sequence (placement tentative). Included for reference are representatives or close relatives of bacteria reported to be associated with various forms of lobster shell lesions; T indicates type strains. The bar indicates distance values (the number of substitutions as a proportion of the length of the alignment, excluding gaps).

It was possible to identify a small number of bands that were consistently (1) associated with necrotic lesions but absent from healthy, or healed, cuticle (i.e., possible pathogens); or (2) present in healthy cuticle but lost upon lesion formation, or appearing in healing cuticle (i.e., possible beneficial species, or species whose growth was limited among lesion flora). These bands, which appeared to be common denominators for specific cuticle conditions, constituted the focus of further study and were sequenced and assigned tentative identifications based on 16S rRNA sequence homology (Table 1). A large proportion of the selected bacteria belonged to the *γ*-proteobacteria, irrespective of the health or origin of the cuticle (Table 1, Figs. 4, 5). Several closely related, but separate, species belonging to the genus *Arenicella* (*γ*-proteobacteria) were identified, particularly from the lesions of both American and European lobsters. Seven TTGE bands with different migration coefficients (Rf) values were identified as separate members of the *Arenicella* genus, with two found on damaged, but apparently healthy cuticle, and five being found exclusively in lesions. Of all the bacterial species isolated from lesions, only the *Arenicella* species were present at time points corresponding to first detection of the lesion, indicating their possible role in initiating necrosis.

**Table 1:** Bacterial phylotypes identified by culture-independent analysis of carapace and lesion biofilms

Band(s)	NCBI GenBank Accession number	Bacterial division	Genus or closely related genera	Closest BLAST match (% identity, % coverage)	Lobster species	Cuticle sample	*A*. h
E1, A, D	KF631437	*γ*-proteobacteria (incertae sedis)	*Arenicella*	JX455253.1 (99, 100)	*Hg*	Lesion	−
N1, G	KF631439	*γ*-proteobacteria (incertae sedis)	*Arenicella*	JX455253.1 (99, 100)	*Hg, Ha*	Lesion^1^	+
P1	KF631440	*γ*-proteobacteria (incertae sedis)	*Arenicella*	JX455253.1 (98, 100)	*Ha*	Healing damage	+
S1, U, V	KF631441	*γ*-proteobacteria (incertae sedis)	*Arenicella*	FM214426.1 (99, 100)	*Ha*	Lesion	−
2b	KF631438	*γ*-proteobacteria (incertae sedis)	*Arenicella*	JX455260.1 (98, 100)	*Hg*	Lesion	−
10	KF631442	*γ*-proteobacteria (incertae sedis)	*Arenicella*	JQ347341.1 (98, 98)	*Hg*	Healing damage	−
D1	KF631443	*γ*-proteobacteria (incertae sedis)	*Arenicella*	JQ287336.1 (98, 100)	*Hg* (UK only)	Lesion	−
B, I	KF631432	*γ*-proteobacteria	*Eionea*	JQ179027 (96, 100)	*Hg*	Lesion	+
9	KF631435	*γ*-proteobacteria (incertae sedis)	Unclassified *γ*-proteobacterium	HM134446 (98, 99)	*Hg*	Healing damage	−
L3	KF631433	Unclassified *γ*-proteobacteria (incertae sedis)	Unidentified gut bacterium of *Nephrops norvegicus*	JN092286.1 (100, 100)	*Hg, Ha*	Healing damage	−
M2	KF631436	Flavobacteriaceae	*Maribacter*	JN092226.1 (98, 100) Unidentified gut bacterium of *Nephrops norvegicus*	*Hg, Ha*	Lesion^1^	+
13^2^	KF631434	Flavobacteriaceae	Unclassified Flavo bacteriaceae	KC006315.1 (97, 97)	*Hg, Ha*	Healthy^2^	−
7^2^	Length below GenBank minimum	*α*-proteobacteria	Unclassified *α*-proteobacterium	JQ661097 (100, 100)	*Hg, Ha*	Healthy^2^	−

Identities are based on sequence affiliations for 16S rRNA partial gene sequences arising from cloned TTGE bands-of-interest, matched to NCBI Genbank and Ribosomal Database Project entries. *A*. h = *Aquimarina* sp. ‘homaria’ (presence +, or absence −, determined by specific PCR). *Ha, Homarus americanus*; *Hg, Homarus gammarus*.

1 Including cases of spontaneous lesions.

2 Band disappears later upon lesion formation.

Other bacteria that were associated exclusively with necrotic lesions (being absent from healthy cuticle or healed cuticular damage) were members of the genus *Eoina* (*γ*-proteobacteria; found in European lobster lesions only) and the genus *Maribacter* (Flavobacteriaceae). It is also of note that both these bacteria were only detected in lesions containing *A*. sp. ‘homaria’. In addition to the two bacteria identified as members of *Arenicella*, a further two *γ*-proteobacteria were associated specifically with healing cuticle (Table 1, Fig. 4). Two bacterial species were exclusively associated with healthy cuticle from all the lobster cohorts, being absent from lesions (Table 1, Fig. 4). One bacterium (from band 7) belonged to the *α*-proteobacteria, order Rhizobiales, while the other bacterium had closest homology to the Flavobacteriaceae but appears to be a novel species and cannot currently be assigned to a particular order.

Swabs of aquarium tank walls and plastic containers in which the lobsters were housed, were also analyzed by TTGE. In these samples, the mean number of bacterial species resolved as discrete bands by TTGE was 7.9 (±SE 0.7). The banding patterns (i.e., numbers of bands and their position on the gel) appeared less diverse among these samples, with several common bands shared between the samples from different tank substrates (Fig. S6). Based on band migration coefficients (Rf values), however, none of these samples shared banding profiles with those specific to lobster cuticle lesions. Consequently, no bands were selected for further analysis.

### Prevalence of *Aquimarina* sp. ‘homaria’

**Figure 6:**
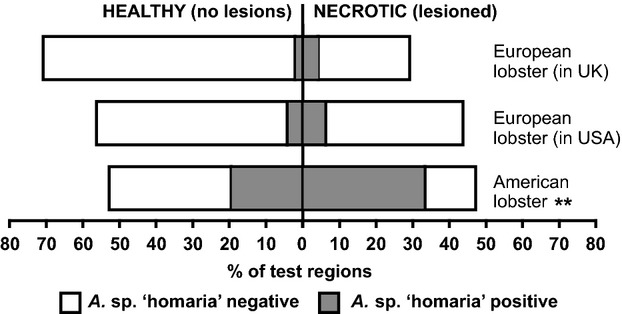
Prevalence of *Aquimarina* sp. *‘*homaria’ in swabs taken from both lesioned and healthy lobster cuticle samples. *Aquimarina* sp. ‘homaria’ was detected by PCR and a sample was classified as positive if the bacterium was detected at any point during the study. Experimentally damaged and control regions are combined. A much greater proportion of lesions were associated with *A*. sp. ‘homaria’ among the American lobsters than in either cohort of European lobster (**P < 0.01). The bacterium was also more prevalent in healthy cuticle samples taken from American lobsters versus European lobsters. All the environmental swabs tested negative for *A*. sp. ‘homaria’.

None of the cuticle samples were *A*. sp. ‘homaria’ positive by PCR at time zero. However, as the experiment progressed, this bacterium was detected in several cuticle swabs, and with a higher prevalence among American lobsters than among either of the European lobster cohorts (Fig. 6). In American lobsters, 78% of the abraded carapace samples and 78% of the damaged claw samples tested positive for *A*. sp. ‘homaria’ at some point during the experiment, and *A*. sp. ‘homaria’ was also detected in 22% of the carapace control regions and 33% of the control regions from claw samples. In contrast, European lobsters held in the United States had a prevalence of 17% (abraded carapace) and 25% (damaged claw), and European lobsters held in the United Kingdom had a prevalence of just 8% (for both abraded carapace and damaged claw samples). None of the control regions sampled in the European lobsters tested positive for *A*. sp. ‘homaria’. The higher prevalence of *A*. sp. ‘homaria’ in American compared with European lobsters was statistically significant (carapace: *P* = 0.0012, *x*^2^ = 13.4, df = 2; claw: *P* = 0.0028, *x*^2^ = 11.75, df = 2) but there was no significant difference in *A*. sp. ‘homaria’ prevalence between the two separate European lobster cohorts held in the two separate locations.

When considering all American lobster lesions combined (i.e., both damaged and control regions), 71% were *A*. sp. ‘homaria’ positive. The overall percentage of European lobster lesions that were *A*. sp. ‘homaria’ positive was lower, and was identical for both separate cohorts (14%). The presence of *A*. sp. ‘homaria’, however, was not significantly correlated with lesion formation (*P* = 0.0543, Fisher's exact test), with many lesions forming before detection of the bacterium. Conversely, *A*. sp. ‘homaria’ was sometimes detectable on damaged and control regions that showed no signs of concurrent or later lesion formation (for further details, see Table S2). The anatomical distribution of *A*. sp. ‘homaria’ was relatively consistent, with equal or close to equal prevalences between claw and carapace, irrespective of lobster species (data not shown). *Aquimarina* sp. ‘homaria’ was not detectable by PCR in any of the environmental biofilm samples at any time point, in either aquarium system.

### Overall body-wide health of the cuticle

As detailed in Data S1, observations of the entire body surface over several months suggested that American lobsters exhibited fewer spontaneous dorsal and periopod lesions than the European lobsters. Additionally, European lobsters held in the United Kingdom developed more ventral lesions than either of the other two lobster cohorts (see Fig. S7 for details).

## Discussion

A major aim of the study was to determine if the close proximity of American lobsters would prejudice the cuticle health of European lobsters in captivity. In terms of claw damage and risk of shell lesions, it appears that European lobsters may be rendered more susceptible when located in the same aquarium as American lobsters, although the same was not true of carapace abrasion injuries in the experiment. European lobsters also had a higher distribution of lesions over their body surfaces, and such gross differences may reflect self-inflicted damage behaviors at a species-specific level. However, the small sample size versus the number of possible influential parameters (such as roughness of plastic substrates or effects of heterospecific pheromones on behavior) complicate interpretation of these results.

The TTGE analysis of cuticle swabs created an impression of dynamic bacterial communities with variation between individual lobsters. Although 16S metagenomics and denaturing gradient gel electrophoresis analyses of American lobster lesions have been performed before (e.g., those of impoundment shell disease, ESD and enzooitic shell disease (EnSD); Chistoserdov et al. 2012), the present study is the first to use TTGE to compare material from European and American lobsters over a time course spanning the whole of experimentally induced lesion development. The TTGE banding patterns indicated members of three bacterial genera that were specifically associated with cuticular lesions: *Arenicella, Eionea* (in European lobsters only), and *Maribacter*. The *Maribacter* species was most closely related to an unidentified bacterium isolated from the gut of the Norway lobster, *Nephrops norvegicus* (Meziti et al. 2012). *Maribacter* species have previously been associated with lobster shell lesions: Quinn et al. (2012) identified the bacterium *Maribacter polysiphoniae* in spontaneously occurring lesions from captive American lobsters, and Chistoserdov et al. (2012) also reported unique *Maribacter* species in EnSD lesions. The only record of an *Eionea* species occurring on crustaceans was reported recently by Hazra et al. (2013) from healthy carapaces of green, spider and Atlantic horseshoe crabs (*Carcinus maenas, Libinia emarginata* and *Limulus polyphemus*, respectively). There are no reports of these bacteria being associated with lobsters, nor are there any published reports of *Arenicella* sp. being identified from lobster samples. *Arenicella xantha* was originally found in marine sandy sediments (Romanenko et al. 2010), and it is also interesting that *Arenicella* species, according to BLAST 16S rRNA homologies, are closely related to symbionts of marine worms and bivalves. It is, therefore, plausible that the bivalve component of the lobster diet could be a source of *Arenicella*, although this was not investigated.

In this study, claw wounds from several lobsters transiently harbored nematodes, copepods, and polychaete worms, and these were often (though not exclusively) associated with lesions. It is likely that epibionts or parasites are attracted to and feed on necrotic tissue and bacteria, but it is unknown whether the relationship could be beneficial to the lobster (as in maggot debridement therapy in human medicine) or exacerbating. Our attempts to capture and extract bacterial DNA from epibionts failed, but it could be hypothesized that some of the bacterial species represented in our swabs samples originated from parasites.

Two TTGE bands were consistently found in healthy cuticle samples from all the lobster cohorts, which then disappeared upon lesion formation. Phylogenetic analysis of band 13 revealed close homology to the Flavobacteriaceae, including, paradoxically, some species that are known degraders of polysaccharides (e.g., *Lacinutrix* sp.). The second band (band 7) unfortunately yielded only a short sequence so that few reliable inferences can be made about its taxonomy. It is tentatively assigned to the *α*-proteobacteria, most likely a member of the order Rhizobiales, which contains numerous symbiotic and beneficial species. It is of significance that Bell et al. (2012) also reported that members of the Rhizobiales were strongly represented in the normal flora of American lobsters.

Bacterial genera correlated with American lobster ESD were not detected in any of the bands selected from our TTGE analysis (e.g., *Jannaschia*; Meres et al. 2012). Notable too for their absence were members of the genus *Vibrio*, which have been strongly implicated in non-ESD forms of shell disease in crabs (Vogan et al. 2002; Hazra et al. 2013), but not in American lobsters (Chistoserdov et al. 2012; Meres et al. 2012) except possibly in cases of trauma shell disease (Quinn et al. 2013). Meres et al. (2012) demonstrated that the abundance of *α*-proteobacteria, *γ*-proteobacteria and Bacteroidetes increased on diseased American lobster cuticles, while flavobacteria decreased. While this study did not have sufficient coverage to make the same comparisons, it was interesting to note that all but one of the bacterial species identified from lesions belonged to the *γ*-proteobacteria while no *γ*-proteobacteria were represented in the bands exclusively associated with healthy cuticle.

Although *A*. sp. ‘homaria’ was detected on many occasions, and particularly on American lobsters, its role as an initiator of lesion formation in this study is doubtful because it rarely appeared until after lesions were established. It was also sometimes detected on healthy cuticle sample that subsequently remained healthy for several months. One animal (European lobster 11EU026 held in Boston) exhibited large areas of rapidly spreading, coalescing lesions across the dorsal carapace. Although a diagnosis of the type of shell disease could not be made due to the lack of histological examination, these lesions also consistently tested negative for *A*. sp. ‘homaria’. Our data suggest that in normal shell disease, and especially in European lobsters, *A*. sp. ‘homaria’ was an opportunistic species that probably exacerbated the disease process rather than initiating it. It remains unclear why its prevalence was so much higher among American lobsters, but striking differences in shell morphology might be a contributory factor (Davies et al. 2014). It was also interesting to note that being housed with American lobsters for a prolonged period did not result in increased prevalence of *A*. sp. ‘homaria’ among the European lobsters in this study. This in turn suggested that the bacterium was not easily transmitted between the two lobster species. Furthermore, among the bacteria detected from European lobsters, none were specific only to the individuals held in the Boston aquarium, which suggests a lack of heterospecific bacterial transmission. It remains to be seen whether this would still be true if the lobsters were allowed direct physical contact, but given the potential for serious injury such experiments were not performed.

For the duration of the study, none of the lesions in either lobster species healed once necrosis had become established (the healing process, when present, began soon after wounding). Molting is another strategy available to resolve lesions, and it is unsurprising that some lobsters (13% of European lobsters and 30% of American lobsters) molted during a 5–7 week period after wounding. The many cases of unrelenting necrosis suggested that the defenses of these lobsters were overwhelmed locally. The involvement of a localized (or indeed, systemic) immune response, other than melanization, was beyond the scope of this study but obviously deserves investigation.

## Supporting Information

**Data S1.** Supporting experimental procedures. **Figure S1.** Lack of correlation between time since last molt versus the risk of lesion formation following experimental damage to claw or carapace. A, B, C = carapace; D, E, F = claw. Time zero refers to the start of the experiment. **Figure S2.** Lack of correlation between duration of intermolt period and risk of lesion formation in control regions of carapace and claw. A, B, C = carapace; D, E, F = claw. Time zero refers to the start of the experiment. **Figure S3.** Lack of correlation between lobster size and the risk of lesion formation during the study period. Lobster size is represented by carapace length. Time zero refers to the start of the experiment. **Figure S4.** Lobster cuticle lesion morphological types. The relative abundance of each lesion morphological type in experimentally damaged cuticle regions of American lobsters, and European lobsters held in the United States or United Kingdom. Several of the abraded carapace regions exhibited more than one kind of lesion. **Figure S5.** Lesion morphology and distribution on the dorsal cuticle and claw of European lobster EU026. Top row (A) = progressive necrosis of experimentally abraded carapace over 12 weeks. Left panel (B) = spontaneous necrosis of unabraded abdominal segment. Right panel (C) = spontaneous lesions adjacent to experimentally induced claw damage (arrow). Bottom row (D) = unusual spontaneous necrosis of unabraded carapace. Note the large areas of abnormal pigmentation preceding lesion formation, the round, shallow lesions, protruding flesh, and the rapidly spreading, coalescing nature of the lesions. This lobster was maintained in the U.S. aquarium. Lesion and pre-lesion samples from the abraded carapace and damaged claw consistently tested negative for *Aquimarina* sp. ‘homaria’. Bar = 5 mm. **Figure S6.** Examples of TTGE gel banding patterns for bacterial 16S rRNA amplified from environmental swabs and filtered water samples. Swabs were taken from aquarium tank walls in both the Swansea (United Kingdom) and Boston (United States) aquaria, both at the start of the experiment and at the final time point (f). SW = filtered seawater; m = commercial marker lane for gel alignment. Composite image. **Figure S7.** Total-body cuticle health estimates for American and European lobsters. American lobsters (Ha) and European lobsters (Hg) were observed fortnightly for the presence of spots, lesions, and damage over the entire body surface. As an approximate gauge of cuticle health, the maximum percentage of body areas exhibiting lesions was calculated for each lobster (regardless of when that number occurred during the experiment). The dorsal surface (A), ventral surface (B) and periopods (C) were analyzed separately. Horizontal bars represent median values. Stars denote significance values: **P* < 0.05, ***P* < 0.01; ****P* < 0.001. **Table S1.** Record of individual lobster sizes, molt status, and duration of participation in the abrasion/impact damage study. The table indicates all American (*H. americanus*) and European (*H. gammarus*) lobsters used for carapace abrasion and claw impact damage experiments. Each lobster had a unique identification (ID) number. Lobster size is indicated as the carapace length (CL) at the time of shipping European lobsters to the United States. The duration of participation in the abrasion/impact damage experiment is indicated for each lobster. Most animals participated for the full duration of the study, but some were recruited to the study later due to the cuticle being too soft from a recent molt. Some lobsters were dropped out of the study early due to molting. One lobster died shortly before the conclusion of the experiment. See also Figures S1–S3 (above), which show that the risk of lesion formation was not significantly correlated with carapace length or the amount of time since the last molt. **Table S2.** Lack of correlation between *Aquimarina* sp. ‘homaria’ positive cuticle samples and lesion formation. The table indicates all American (*H. americanus*) and European (*H. gammarus*) lobster cuticle samples testing positive for *A*. sp. ‘homaria’ by PCR, the time point(s) at which the bacterium was detected, and whether a lesion developed in the sampled area. Few samples exhibited the presence of *A*. sp. ‘homaria’ concomitant with lesion formation, and many samples remained healthy for the duration of the experiment despite the presence of the bacterium. CL = Claw; CA = carapace; da = damage (crack or puncture); ab = abrasion; con = control (undamaged). Asterisks indicate swabs that were only tested at a single time point.
