# Supplementary material for: Cuticles of European and American lobsters harbor diverse bacterial species and differ in disease susceptibility
Source: Microbiologyopen. 2014 May 12;3(3):395–409. doi: 10.1002/mbo3.174 (PMC4082712; doi:10.1002/mbo3.174)
Supplement: Supplementary file 1 — Data S1. Supporting experimental procedures. Figure S1. Lack of correlation between time since last molt versus the risk of lesion formation following experimental damage to claw or carapace. A, B, C = carapace; D, E, F = claw. Time zero refers to the start of the experiment. Figure S2. Lack of correlation between duration of intermolt period and risk of lesion formation in control regions of carapace and claw. A, B, C = carapace; D, E, F = claw. Time zero refers to the start of the experiment. Figure S3. Lack of correlation between lobster size and the risk of lesion formation during the study period. Lobster size is represented by carapace length. Time zero refers to the start of the experiment. Figure S4. Lobster cuticle lesion morphological types. The relative abundance of each lesion morphological type in experimentally damaged cuticle regions of American lobsters, and European lobsters held in the United States or United Kingdom. Several of the abraded carapace regions exhibited more than one kind of lesion. Figure S5. Lesion morphology and distribution on the dorsal cuticle and claw of European lobster EU026. Top row (A) = progressive necrosis of experimentally abraded carapace over 12 weeks. Left panel (B) = spontaneous necrosis of unabraded abdominal segment. Right panel (C) = spontaneous lesions adjacent to experimentally induced claw damage (arrow). Bottom row (D) = unusual spontaneous necrosis of unabraded carapace. Note the large areas of abnormal pigmentation preceding lesion formation, the round, shallow lesions, protruding flesh, and the rapidly spreading, coalescing nature of the lesions. This lobster was maintained in the U.S. aquarium. Lesion and pre-lesion samples from the abraded carapace and damaged claw consistently tested negative for Aquimarina sp. ‘homaria’. Bar = 5 mm. Figure S6. Examples of TTGE gel banding patterns for bacterial 16S rRNA amplified from environmental swabs and filtered water samples. Swabs were taken from aquarium tank wa [file mbo30003-0395-sd1.docx]

**Supporting Information**

*Whitten et al. Cuticles of European and American lobsters harbor diverse bacterial species and differ in disease susceptibility*

**SUPPORTING EXPERIMENTAL PROCEDURES**

**Aquarium water**

The pH and temperature of the water in both aquarium systems was monitored to ensure similar conditions (pH7.8-8.0; temperature declined naturally with the autumn season from 14°C at the start of the experiment to 10.5°C at its conclusion). Salinity remained very stable in both aquaria, at an average of 30.5 ppt in Boston and 32 ppt in Swansea, fluctuating +/- 0.9 ppt.

**Optimization of bacterial DNA extraction from biofilm samples**

In preliminary experiments, various mechanical and chemical / enzymatic extraction methods were compared for 95% ethanol-preserved biofilm swabs and biofilm samples dried onto FTA® cards (Whatman International Ltd, Maidstone, UK). The aim was to select a method that combined a high yield efficiency with the widest possible phylogenetic diversity of extracted bacterial templates to be amplified and detected by subsequent PCR, while avoiding extraction bias to a particular type of bacteria. Each method was examined using both agarose and TTGE gels. Several universal 16S rRNA primers were also evaluated for the best coverage and band separation by TTGE. Three pre-treatment extraction methods were compared for subsequent processing with each of two commercially available kits: (1) digestion in 0.05 mg/ml lysozyme with 0.5M EDTA, pH 8 (37°C, 1 hr), followed by incubation in RNase-supplemented Nuclei Lysis Solution (Promega) (80°C, 10 mins); (2) digestion in 0.05 mg/ml lysozyme with 0.5M EDTA, pH 8 (37°C, 1 hr), followed by proteinase K digestion (55°C, 1 hr); (3) incubation in RNase-supplemented Nuclei Lysis Solution (Promega) (80°C, 10 mins). Samples were then processed with either the Wizard SV Genomic DNA purification kit (Promega) or the Qiagen DNeasy**®** Blood and Tissue Kit. FTA® cards were treated in FTA purification reagent then washed in TE buffer (10mM Tris, 0.1M EDTA, pH 8) for direct use in PCR reactions.

The optimal method chosen for the main study was extraction from swabs, using a modification of the Qiagen DNeasy**®** Blood and Tissue Kit, incorporating pre-treatment step (2) as above, i.e. initial lysozyme-based disruption step optimized for Gram-positive bacteria, followed by proteinase K digestion.

## Cloning of TTGE bands-of-interest

Excised TTGE gel bands were purified using the Wizard® Preps PCR DNA purification system (Promega) optimized for urea-rich polyacrylamide gels. The purified DNA was ligated into a pGEMTeasy cloning vector (Promega) then transformed into *E. coli* XL1Blue cells by heat-shock. Positive transformants were identified by blue / white selection and antibiotic resistance. Minipreps from up to six colonies per cloned TTGE band were sequenced. To confirm that the cloned fragments reflected the original band of interest, the minipreps were used as templates for PCR using PRBA338f (GC-clamped) and PRUN518r primers and each product was screened against the original swab sample by an additional TTGE.

**Effect of molt interval and** **carapace length (CL) on risk of lesion formation**

The lobsters in this study were abraded and damaged at varying time points during their molt cycles, ranging between 17 and 100 days post-molt at the start of the experiment (time zero). Molt intervals (i.e. the time elapsed since the last molt) were not able to be controlled due to experimental time constraints.

Similarly, the lobsters varied in size, and the average carapace length (CL) of European lobsters held in Swansea was greater than that of European lobsters held in Boston. Since shell disease progresses over time and often worsens just prior to a molt (Tlusty and Metzler, 2012), it was necessary to determine whether correlations existed between (i) a lobster’s stage in its molt cycle and the risk of formation of lesions on freshly damaged carapace and claw, and (ii) whether the CL correlated with lesion emergence. Frequency distributions of (i) the post-molt intervals and (ii) the CL for each lobster were plotted against the time of lesion emergence during the experiment, and analysed by the Spearman correlation analysis. No significant correlations were identified between molt cycle timing and risk of lesion formation (**Fig S1**; American lobsters r_s_=0.397, 2-tailed P=0.291 for abraded carapace and r_s_=0.1895, 2-tailed P=0.613 for damaged claw; European lobsters in USA r_s_=-0.414, 2-tailed P=0.20 for abraded carapace and r_s_=-0.1334, P=0.694 for damaged claw; European lobsters in UK r_s_=0.150, 2-tailed P=0.635 for abraded carapace and r_s_=-0.344, 2-tailed P=0.276 for damaged claw). Similarly, no correlation between molt cycle timing and risk of lesion formation existed in the control regions of the cuticle (Spearman correlation analysis: American lobsters r_s_=0.307, 2-tailed P=0.410 for control carapace and r_s_=0.131, 2-tailed P=0.744 for control claw; European lobsters in USA r_s_=0.027, 2-tailed P=0.946 for control carapace and r_s_=-0.410, P=0.203 for control claw; European lobsters in UK r_s_=0.131, 2-tailed P=0.685 for control carapace and r_s_=0.054, 2-tailed P=0.868 for control claw; **Fig S2**). It was therefore concluded that age of the cuticle was not a significant predictor or risk factor for lesion formation in new areas of damage (unless that area overlapped an already-diseased part of the shell), and that the differences in molt cycle timing would not confound subsequent analyses in our study.

No significant correlations were identified between CL and risk of lesion formation (**Fig S3**; American lobsters r_s_=0.447, 2-tailed P=0.230 for abraded carapace and r_s_=0.00, 2-tailed P=1.012 for damaged claw; European lobsters in USA f r_s_=0.380, 2-tailed P=0.250 for abraded carapace and r_s_=0.17, 2-tailed P=0.618 for damaged claw; European lobsters in UK r_s_=0.315, 2-tailed P=0.318 for abraded carapace and r_s_=0.306, 2-tailed P=0.333 for damaged claw). Similarly, no correlation between CL and risk of lesion formation existed in the control regions of the cuticle (Spearman correlation analysis: American lobsters r_s_=-0.481, 2-tailed P=0.194 for control carapace and r_s_=-0.663, 2-tailed P=0.059 for control claw; European lobsters in USA r_s_=0.0.00, 2-tailed P=1.00 for control carapace and r_s_=0.405, 2-tailed P=0.217 for control claw; European lobsters in UK r_s_=0.481, 2-tailed P=0.113 for control carapace and r_s_=0.256, 2-tailed P=0.422 for control claw; **Fig S3**). It was also concluded, therefore, that the size differences of the lobsters used in this study did not have a significant impact on the risk of lesion development.

**Whole body cuticle health survey**

Each lobster was visually inspected fortnightly and was conducted preceeding, during, and after the experimental damage study for a maximum of 195 days (approximately 28 weeks). As described in Tlusty and Metzler (2012), early stages of shell disease exhibit as melanized areas, or spots, but without visible shell erosion. As disease progresses, noticeable shell erosion, or lesions, can occur, and are the typical symptoms associated with shell disease. Lobsters were inspected for the presence of spots and lesions over the entire cuticle surface. Photographic records were kept on a fortnightly basis to assess damaged and control carapace, as well as spontaneous lesions and other interesting features (e.g. epibiont shell fouling, limb loss, injury, molts, visible evidence of melanized nodules in underlying tissues).

The body surface was sub-divided into 123 areas and, as an approximate index of cuticle health, the maximum percentage of body areas exhibiting lesions was calculated for each lobster, regardless of when that number occurred during the experiment (Tlusty and Metzler, 2012). The dorsal surface, ventral surface and periopods were analysed separately since 2-way ANOVA indicated the data were unsuitable for pooling. Periopod data were abnormally distributed and were analysed by 1-way Kruskal-Wallis ANOVA with Dunn’s multiple comparisons post-test. Dorsal and ventral data were each analysed by parametric 1-way ANOVA with Tukey’s multiple comparisons post-test. American lobsters exhibited fewer dorsal and periopod lesions than the European lobsters. Additionally, European lobsters held in the UK had more ventral lesions than either of the other two lobster cohorts (See **Fig.** **S7** for details). These gross differences are possibly indicative of behavioral differences between the lobster species.

**SUPPORTING REFERENCES**

Tlusty, M.F., and Metzler, A. (2012) Relationship between Temperature and Shell Disease in Laboratory Populations of Juvenile American Lobsters (*Homarus americanus*). J Shellfish Res 31: 533-541.

**SUPPORTING FIGURES**

**Fig S1**

**Lack of correlation between time since last molt versus the risk of lesion formation following experimental damage to claw or carapace.** A, B, C = carapace; D, E, F = claw. Time zero refers to the start of the experiment.


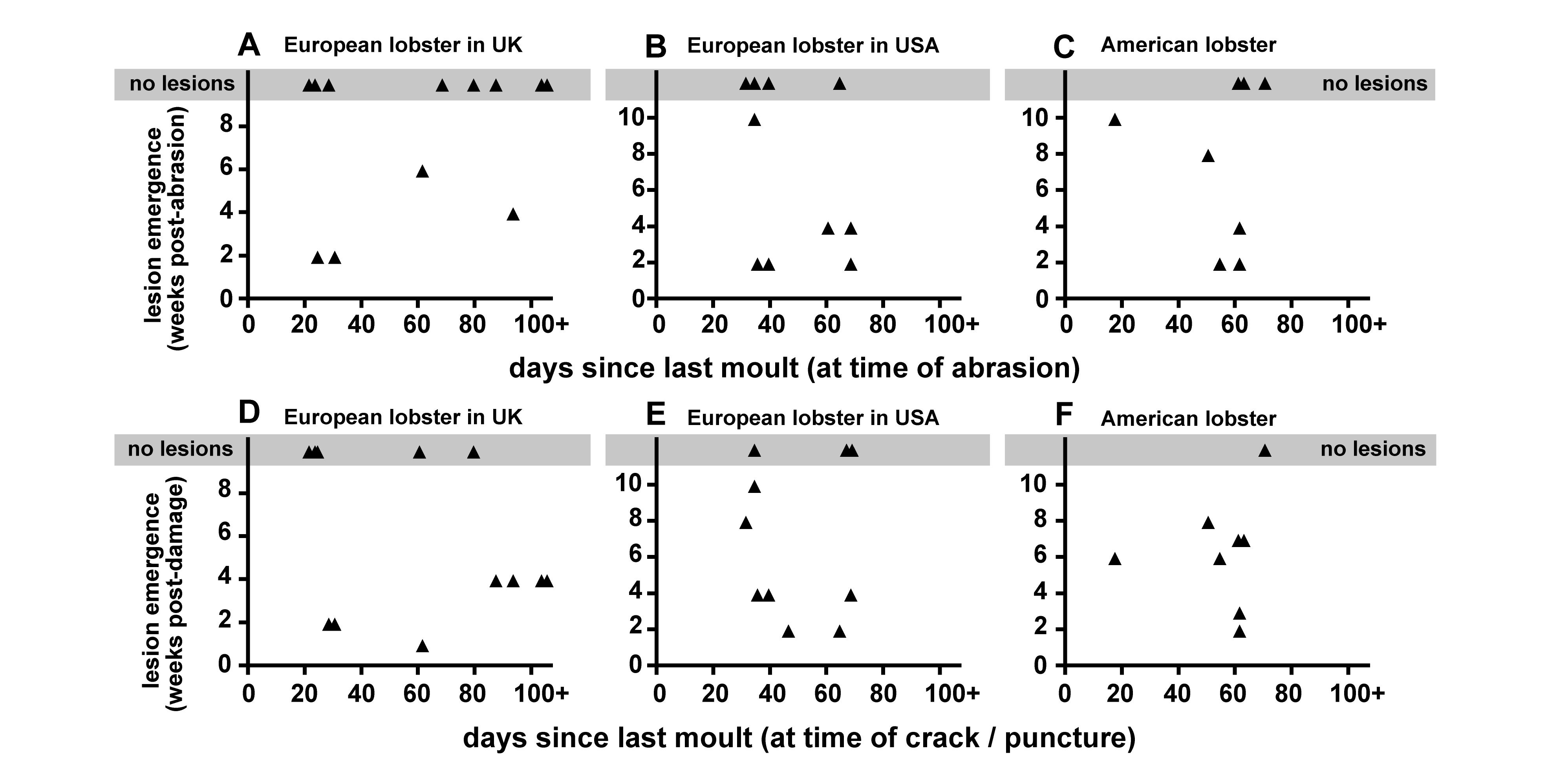


**Fig S2**

**Lack of correlation between duration of intermolt period and risk of lesion formation in control regions of carapace and claw.** A, B, C = carapace; D, E, F = claw. Time zero refers to the start of the experiment.


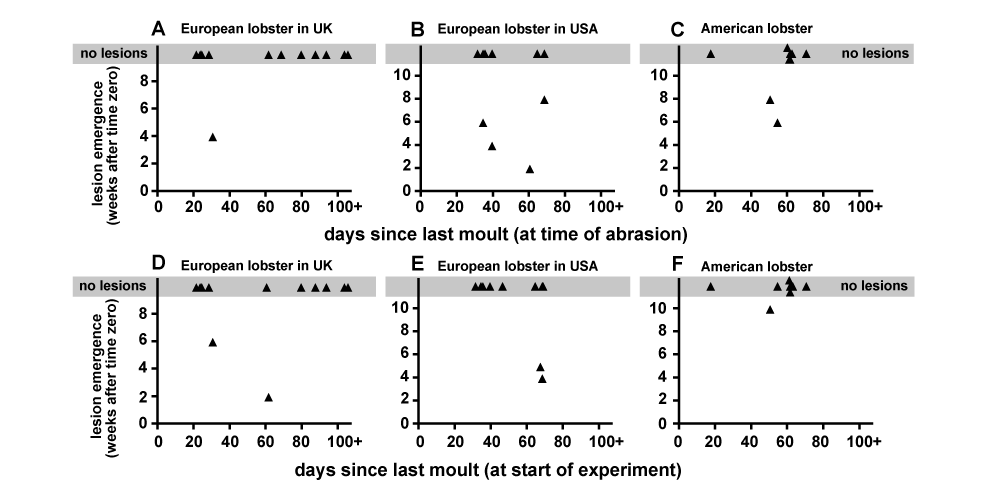


**Fig S3**

**Lack of correlation between lobster size and the risk of lesion formation during the study period.** Lobster size is represented by carapace length. Time zero refers to the start of the experiment.


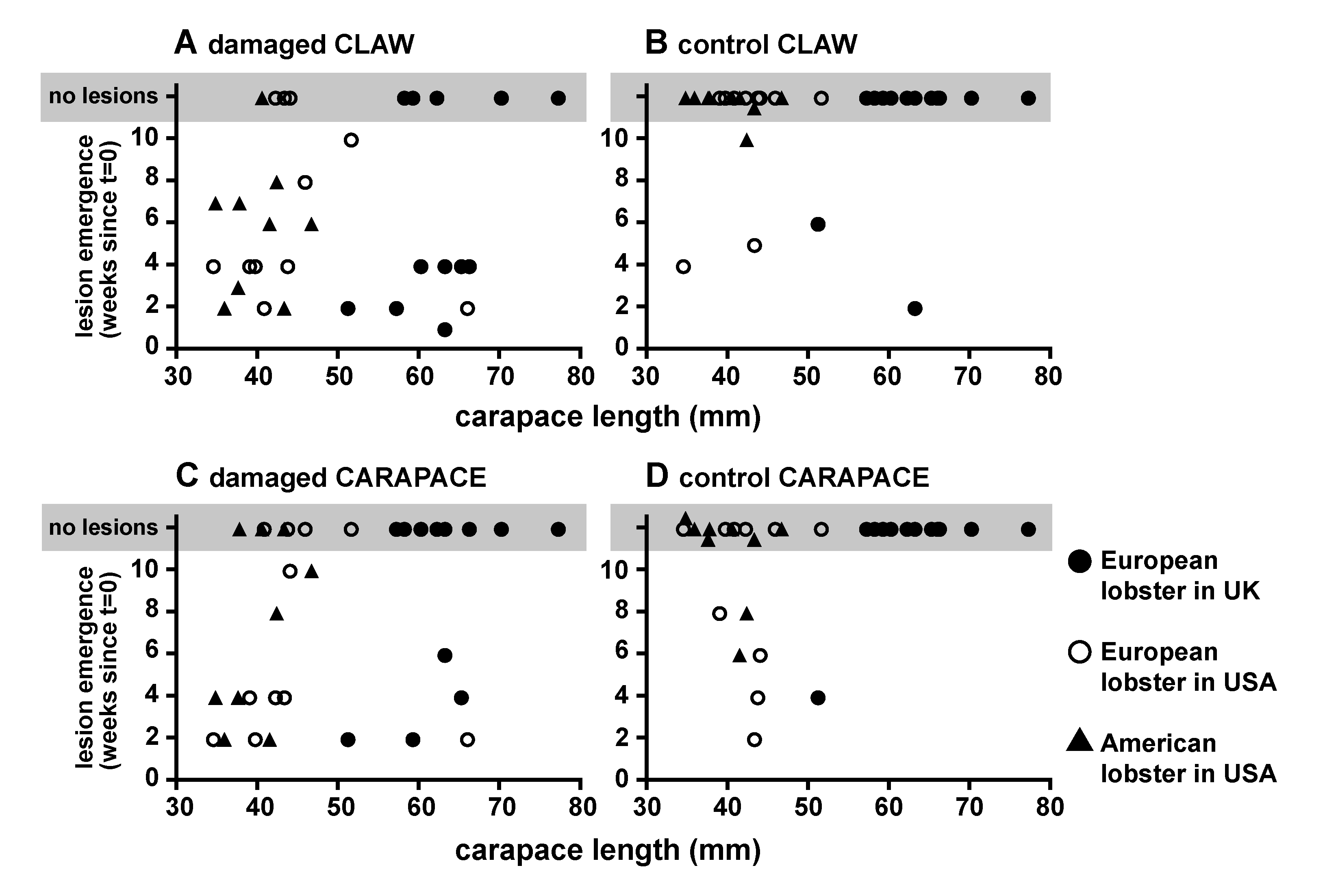


**Fig S4**

**Lobster cuticle lesion morphological types.** The relative abundance of each lesion morphological type in experimentally damaged cuticle regions of American lobsters, and European lobsters held in the USA or UK. Several of the abraded carapace regions exhibited more than one kind of lesion.


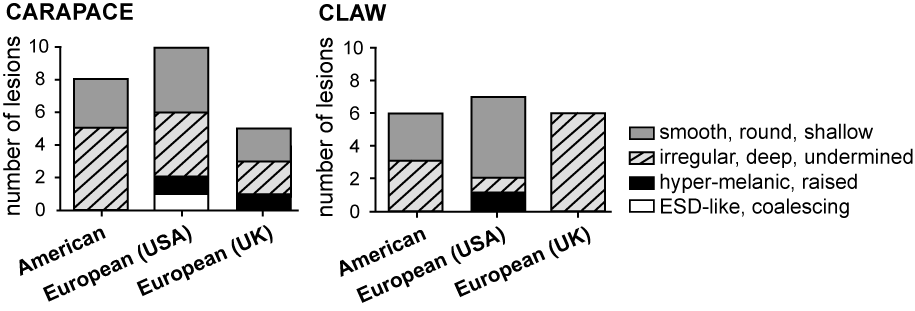


**Fig S5**

**Lesion morphology and distribution on the dorsal cuticle and claw of European lobster EU026.** Top row (A) = progressive necrosis of experimentally abraded carapace over 12 weeks. Left panel (B) = spontaneous necrosis of unabraded abdominal segment. Right panel (C) = spontaneous lesions adjacent to experimentally induced claw damage (arrow). Bottom row (D) = unusual spontaneous necrosis of unabraded carapace. Note the large areas of abnormal pigmentation preceding lesion formation, the round, shallow lesions, protruding flesh, and the rapidly spreading, coalescing nature of the lesions. This lobster was maintained in the USA aquarium. Lesion and pre-lesion samples from the abraded carapace and damaged claw consistently tested negative for *Aquimarina* sp. ‘homaria’. Bar = 5 mm.


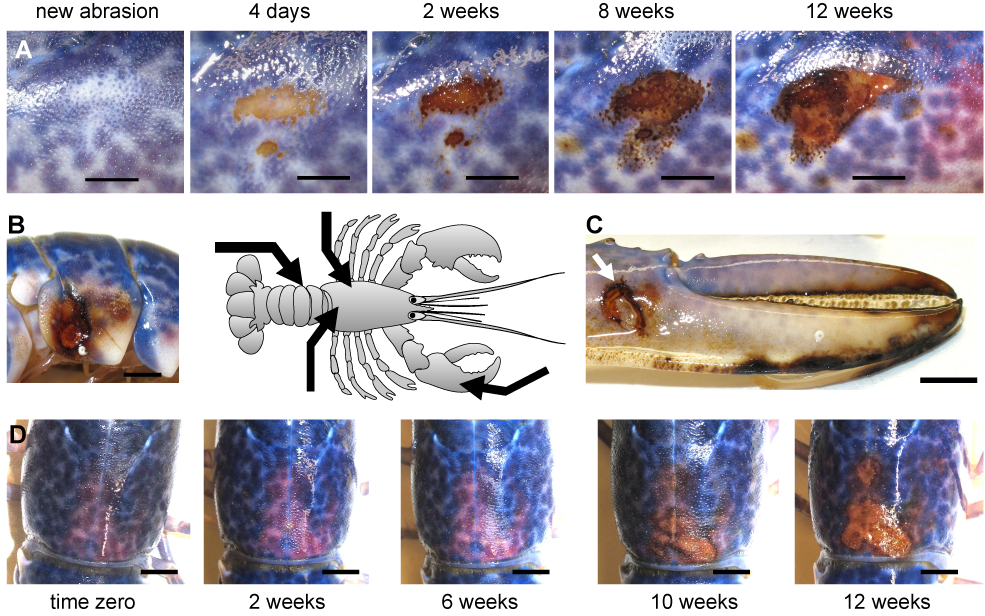


**Fig S6**

**Examples of TTGE gel banding patterns for bacterial 16S rRNA amplified from environmental swabs and filtered water samples.** Swabs were taken from aquarium tank walls in both the Swansea (UK) and Boston (USA) aquaria, both at the start of the experiment and at the final time point (f). SW = filtered seawater; m = commercial marker lane for gel alignment. Composite image.


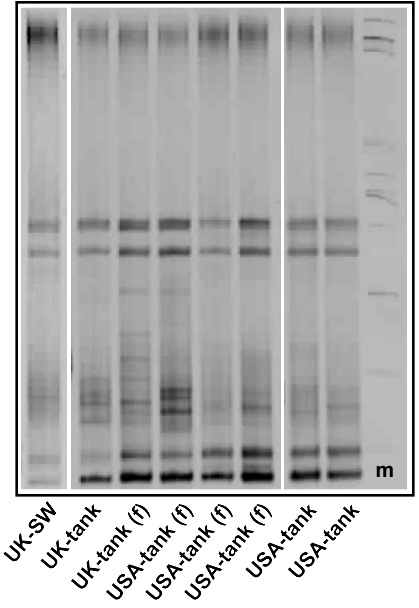


**Fig S7**

**Total-body cuticle health estimates for American and European lobsters.** American lobsters (Ha) and European lobsters (Hg) were observed fortnightly for the presence of spots, lesions and damage over the entire body surface. As an approximate gauge of cuticle health, the maximum percentage of body areas exhibiting lesions was calculated for each lobster (regardless of when that number occurred during the experiment). The dorsal surface (A), ventral surface (B) and periopods (C) were analysed separately. Horizontal bars represent median values. Stars denote significance values: * = P<0.05, ** = P<0.01; *** = P<0.001.


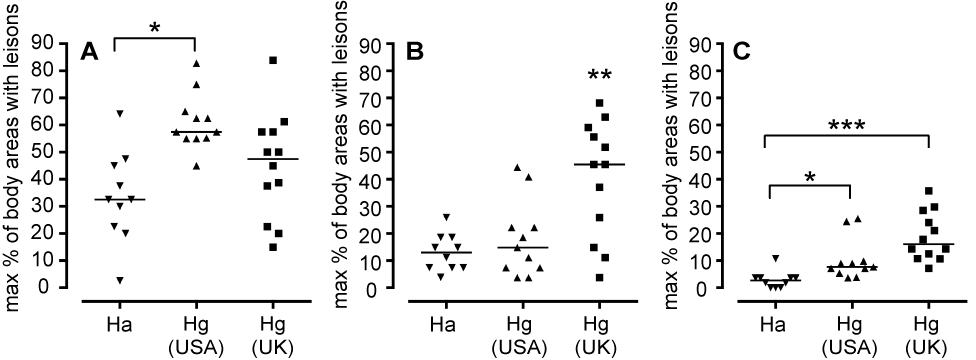


**Table S1**

**Record of individual lobster sizes, molt status and duration of participation in the abrasion / impact damage study.** The table indicates all American (*H. americanus*) and European (*H. gammarus*) lobsters used for carapace abrasion and claw impact damage experiments. Each lobster had a unique identification (ID) number. Lobster size is indicated as the carapace length (CL) at the time of shipping European lobsters to the USA. The duration of participation in the abrasion / impact damage experiment is indicated for each lobster. Most animals participated for the full duration of the study, but some were recruited to the study later due to the cuticle being too soft from a recent molt. Some lobsters dropped out of the study early due to molting. One lobster died shortly before the conclusion of the experiment. See also Figs S1, S2 and S3 (above), which show that the risk of lesion formation was not significantly correlated with carapace length or the amount of time since the last molt.

| ***H. gammarus* held in UK** | | | ***H. gammarus* held in USA** | | | ***H. americanus* held in USA** | | |
| --- | --- | --- | --- | --- | --- | --- | --- | --- |
| **ID** | **CL**  **(mm)** | **Participation in experiment** | **ID** | **CL (mm)** | **Participation in experiment** | **ID** | **CL**  **(mm)** | **Participation in experiment** |
| G1 | 66 | molted after 7 weeks | EU025 | 39 | full | MS016 | 37 | recruited 5 weeks late (too soft) |
| G2 | 59 | recruited 2 weeks late (too soft) | EU026 | 39 | full | MS161 | 42 | full |
| G3 | 60 | full | EU027 | 42 | molted after 7½ weeks | MS186 | 43 | full |
| G4 | 65 | full | EU028 | 39 | full, but recruited 3 weeks late (too soft) | MS011 | 36 | full |
| G5 | 63 | full | EU029 | 34 | full | MS054 | 35 | molted after 7 weeks |
| G6 | 57 | full | EU030 | 37 | full | MS061 | 43 | full |
| O1 | 63 | full | EU031 | 46 | full | MS090 | 37 | molted after 6 weeks |
| O2 | 62 | full | EU032 | 40 | full, but recruited 4 weeks late (too soft) | MS168 | 38 | full |
| O3 | 77 | molted after 5 weeks | EU033 | 38 | full, but recruited 2 weeks late (too soft) | MS042 | 40 | full |
| O4 | 70 | full | EU034 | 45 | full | MS043 | 40 | molted after 5 weeks |
| O5 | 58 | died after 7 weeks | EU035 | 61 | full |  |  |  |
| O6 | 51 | full |  |  |  |  |  |  |

**Table S2**

**Lack of correlation between *Aquimarina* sp. ‘homaria’ positive cuticle samples and lesion formation.** The table indicates all American (*H. americanus*) and European (*H. gammarus*) lobster cuticle samples testing positive for *A.* sp. ‘homaria’ by PCR, the time point(s) at which the bacterium was detected, and whether a lesion developed in the sampled area. Few samples exhibited the presence of *A.* sp. ‘homaria’ concomitant with lesion formation, and many samples remained healthy for the duration of the experiment despite the presence of the bacterium. CL = Claw; CA = carapace; da = damage (crack or puncture); ab = abrasion; con = control (undamaged). Asterisks indicate swabs that were only tested at a single time point.

| **Cuticle region & treatment** | **Time of lesion formation** | **Time of**  ***A.* sp. ‘homaria’ detection** | **Link between**  ***A.* sp. ‘homaria’ & lesion formation?** |
| --- | --- | --- | --- |
| ***H. gammarus* held in the USA:** | | | |
| ab-CA | n/a (healed) | 6wk onwards | *no lesion* |
| ab-CA | n/a (healed) | 8wk onwards | *no lesion* |
| da-CL | 4 wk + | 12wk* | inconclusive |
| da-CL | 4 wk + | 8wk* | inconclusive |
| da-CL | 8 wk + | 8wk | **possible role in lesion formation** |
| ***H. gammarus* held in the UK:** | | | |
| da-CL | 4 wk + | 8wk* | inconclusive |
| ab-CA | n/a (healed) | 7wk* | *no lesion* |
| con-CL | 2 wk + | 4wk | *A.* sp. ‘homaria’ too late to initiate lesion |
| ***H. americanus* held in the USA:** | | | |
| ab-CA | 10 wk + | 12wk* | inconclusive |
| ab-CA | 2 wk + | 8wk* | inconclusive |
| ab-CA | 8 wk + | 10wk onwards | *A.* sp. ‘homaria’ too late to initiate lesion |
| ab-CA | 2 wk + | 6wk onwards | *A.* sp. ‘homaria’ too late to initiate lesion |
| ab-CA | 4 wk + | 6wk* | inconclusive |
| ab-CA | 4 wk + | 2wk onwards | **possible role in lesion formation** |
| ab-CA | n/a (healed) | 5wk | *no lesion* |
| da-CL | 8 wk + | 2wk onwards | **possible role in lesion formation** |
| da-CL | 8 wk + | 2wk onwards | **possible role in lesion formation** |
| da-CL | 6 wk + | 12wk* | inconclusive |
| da-CL | 7 wk + | 8wk* | inconclusive |
| da-CL | 7 wk + | 6wk* | inconclusive |
| da-CL | 3 wk + | 2wk onwards | **possible role in lesion formation** |
| da-CL | n/a (healed) | 2 & 6 wk | *no lesion* |
| con-CA | n/a (healthy) | 6wk | *no lesion* |
| con-CL | n/a (healthy) | 4wk | *no lesion* |
| con-CL | n/a (healthy) | time zero | *no lesion* |
| con-CA | n/a (healthy) | 10wk onwards | *no lesion* |
